# Pilot-Scale Processing and Functional Properties of Antifungal EVOH-Based Films Containing Methyl Anthranilate Intended for Food Packaging Applications

**DOI:** 10.3390/polym14163405

**Published:** 2022-08-19

**Authors:** Alejandro Aragón-Gutiérrez, Raquel Heras-Mozos, Antonio Montesinos, Miriam Gallur, Daniel López, Rafael Gavara, Pilar Hernández-Muñoz

**Affiliations:** 1 Grupo de Tecnología de Materiales y Envases, Instituto Tecnológico del Embalaje, Transporte y Logística, ITENE, Unidad Asociada al CSIC, Calle de Albert Einstein 1, 46980 Paterna, Valencia, Spain; 2 Instituto de Agroquímica y Tecnología de Alimentos, IATA-CSIC, Calle del Catedrático Agustín Escardino Benlloch 7, 46980 Paterna, Valencia, Spain; 3 Instituto de Ciencia y Tecnología de Polímeros, ICTP-CSIC, Calle Juan de la Cierva 3, 28006 Madrid, Spain

**Keywords:** EVOH copolymer, methyl anthranilate, melt-extrusion, film properties, antifungal activity, active packaging

## Abstract

Antimicrobial packaging has emerged as an efficient technology to improve the stability of food products. In this study, new formulations based on ethylene vinyl alcohol (EVOH) copolymer were developed by incorporating the volatile methyl anthranilate (MA) at different concentrations as antifungal compound to obtain active films for food packaging. To this end, a twin-screw extruder with a specifically designed screw configuration was employed to produce films at pilot scale. The quantification analyses of MA in the films showed a high retention capacity. Then, the morphological, optical, thermal, mechanical and water vapour barrier performance, as well as the antifungal activity in vitro of the active films, were evaluated. The presence of MA did not affect the transparency or the thermal stability of EVOH-based films, but decreased the glass transition temperature of the copolymer, indicating a plasticizing effect, which was confirmed by an increase in the elongation at break values of the films. Because of the additive-induced plasticization over EVOH, the water vapour permeability slightly increased at 33% and 75% relative humidity values. Finally, the evaluation of the antifungal activity in vitro of the active films containing methyl anthranilate showed a great effectiveness against *P. expansum* and *B. cinerea*, demonstrating the potential applicability of the developed films for active food packaging.

## 1. Introduction

During the last decades, production, processing, and marketing of fresh food have experimented with considerable changes trying to fit consumers’ demands. However, wastes due to product spoilage and residues from processing (up to 60% of harvested products) are becoming a serious economic and environmental issue [1]. In this regard, packaging is positioned as one of the main tools to reduce food spoilage and, therefore, many efforts from both academia and the food industry are being directed to improve and optimize food packaging systems [2]. Among others, the development of active antimicrobial packaging has emerged as an innovative technique to extend the shelf life by inhibiting microbial growth in foods while maintaining their quality and safety [3,4,5]. This system is based on the deliberate incorporation of components into packaging materials for their later progressive release into the packaged product or the head-space of the food, causing an inhibitory effect against pathogens that affect food products [6]. Thanks to their low costs, wide availability and versatile properties, the research and developments on active packaging systems have been mainly focused on the use of plastic materials [7]. In this line, ethylene vinyl alcohol (EVOH) copolymers are one of the best candidates used in the design and development of active materials intended for food packaging applications. EVOH copolymers, which are synthesized through the hydrolysis of ethylene vinyl acetate (EVA), are characterized by a high transparency, great chemical resistance and outstanding oxygen barrier properties [8,9]. In addition, EVOH based films are recyclable in current infrastructure by regrinding processes and several works also report that EVOH copolymers with up to 44 mol% ethylene content can be degraded in specific environmental conditions and biological media [10,11,12]. Interestingly, its hydrophilic nature, which results in a significant plasticisation of the polymer matrix when it is exposed to high relative humidifies or to food products with high water activity, can be used as the triggering mechanism to allow the release of components from the packaging to the food media [13].

With the aim of providing a more sustainable character to plastic materials and satisfying consumers demands’, the food industry is moving towards the use of innovative and natural ingredients such as essential oils (Eos), which have proven antimicrobial and antioxidant properties with great interest in food packaging applications [14,15]. In this context, we considered to extend the use of methyl anthranilate (MA), a natural ingredient already employed in flavouring foods and drugs, to prepare active packaging films based on EVOH copolymer. Methyl anthranilate is a metabolite responsible of the characteristic odour of Concord grapes and is also present in several essential oils such as neroli, ylang-ylang and jasmine [16]. The current commercial MA is prepared by fossil-based chemical processes since the direct extraction and recovery of methyl anthranilate from the aforementioned sources have been proven to be economically unfeasible due to low yields [17]. However, there are already some works in literature dealing with the fermentative production of MA from renewable sources such as glucose, in order to obtain a biobased natural compound via an eco-friendly route [18].

Nowadays, there are two main routes to incorporate natural active compounds into polymeric matrixes: the direct incorporation of the natural ingredients into the polymer material and encapsulation technologies. On the one hand, encapsulation technologies of active ingredients have been revealed as a very promising strategy. They present numerous advantages such as protecting active compounds from oxidative processes, photodegradation and thermal conditions or providing a controlled release of the compound from the encapsulating agent [19,20]. Pansuwan et al. reported the encapsulation of methyl anthranilate as an essential oil model in polymer microcapsules by employing two different techniques: microsuspension conventional radical polymerization and microsuspension iodine transfer polymerization using methyl methacrylate (MMA) and ethylene glycol dimethacrylate (EGDMA) copolymer as polymer shells [21]. Buendía et al. encapsulated a mixture of Eos composed of carvacrol, oregano and cinnamon in β-cyclodextrins (β-CD) for the development of an active coated cardboard tray [22]. Similarly, Wen et al. encapsulated cinnamon essential oil in β-CD for the manufacturing of an active electrospun nanofibrous film for food packaging applications [23]. Mohammadi et al. investigated the bioactivity of olive leaf extract encapsulated in soybean oil by nano-emulsion technology [24]. Although these studies showed the potential applicability of different technologies to maintain the beneficial properties of bioactives, there is still a gap of knowledge between the use of certain encapsulation strategies and their effective commercial application. In addition, the food manufacturing industry also needs to face various challenges related to the storability and stability of encapsulated bioactive compounds [25,26]. In this regard, the direct incorporation of active components into polymeric matrixes through conventional melt-processing technologies such as extrusion, compounding and injection moulding is still positioned as an explorable and encouraging strategy to prepare active materials scalable in industrial environments. For example, Krzysztof et al. prepared bioactive polypropylene (PP) films incorporated with plasticizers and antimicrobial substances (i.e., oregano oil, rosemary extracted, methylparaben and green tea extract) by cast film extrusion. Authors showed that the presence of plasticizers promoted a gradual release of the active ingredients from the polymer matrix, and thus, the biocidal activity was enhanced [27]. In this study, the effect of plasticizers as release promoters of the active substances can be achieved by tailoring the relative humidity exposure of the bioactive EVOH-based films. More recently, the attention is focused on the development of active materials based on biodegradable polymers. Laorenza et al. produced films of PBAT/PLA blended with carvacrol, citral and α-terpineol essential oils via blown film extrusion [28]. However, the main limitation of these routes is that the high temperatures and the shear forces generated during the process usually result in significant losses of the bioactives due to their degradation or volatilization [29,30,31]. Moreover, none of the previous research worked on the optimization of process conditions to favour the incorporation of active ingredients into the polymer matrixes in the melt state in order to reduce their potential losses. 

Therefore, the objective of this work was to prepare cast-extruded EVOH-based films containing methyl anthranilate as active compound for their use in packaging applications. To this aim, a copolymer composed of 44% ethylene molar content (EVOH_44_) was selected within the EVOH family, since it presents a lower melting temperature due to its crystalline structure and, hence, the extrusion process can be carried out at softer conditions. In addition, a specific screw configuration was designed in order to optimize the melt processing and minimize the potential losses of MA. The influence of different concentrations of methyl anthranilate over the main properties of interest of EVOH films in food packaging applications was intensely studied. Finally, the ability of methyl anthranilate incorporated in the active films to inhibit the growth of *Botrytis cinerea* and *P. expansum* was investigated. 

## 2. Materials and Methods

### 2.1. Materials

Ethylene vinyl alcohol copolymer composed of 44% ethylene molar content was kindly provided by The Nippon Synthetic Chemical Company (Osaka, Japan). Methyl anthranilate (FCC, Food grade) was purchased from Sigma-Aldrich (Sigma-Aldrich Corp., St. Louis, MO, USA). For the microbiological assays, potato dextrose agar (PDA) was purchased from Scharlau (Scharlab S.L., Barcelona, Spain). The fungal strains *Penicillium expansum* (CECT 2278) and *Botrytis cinerea* (CECT2100) were supplied by the Spanish Type Culture Collection (CECT) and were maintained in potato dextrose broth (PDB) with 20% sterile glycerol at −80 °C. The fungal culture was regenerated and maintained by regular subculture at 26 °C on PDA plates.

### 2.2. Pilot Scale Production of Active EVOH-Based Films by Extrusion Processing 

**Figure 1:**
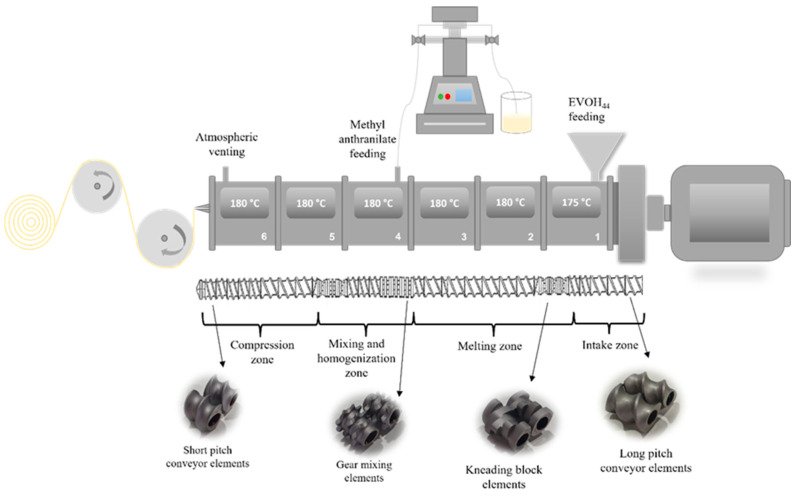
Schematic representation of the film-extrusion process.

Film formulations were prepared in a pilot-scale co-rotating twin screw extruder Brabender TSE 20/40 with a 20 mm diameter screw and a length-diameter ratio (L/D) of 40. EVOH pellets, previously dried at 90 °C for 4 h, were fed through the main hopper employing a gravimetric feeder. On the other hand, a loss-in-weight injection pump was used to feed methyl anthranilate into the polymer melt, taking advantage of its low viscosity. The pumping rate (mL/min) was adjusted for each formulation in order to obtain a final content of MA of 3, 5 and 8 wt.% with respect to the polymer. The active ingredient was fed in the fourth barrel of the extruder with the aim of reducing its residence time and avoid potential losses of the compound associated with its volatile nature. The temperature profile along the 6 barrel zones from hopper (zone 1) to die (zone 6) was 175-180-180-180-180-180 °C (see Figure 1), the applied screw rotation speed was set at 40 rpm, and the throughput was 1.5 kg/h. The employed screw configuration (Figure 1) was designed combining different types of elements (i.e., conveying elements, kneading blocks and gear mixing) in order to achieve a proper mixing and dispersion of the components while preventing a potential thermal degradation of the materials during the extrusion process. In particular, MA was added into gear mixing elements, which have the ability to divide and recombine the flow, promoting a better interaction between the polymer matrix in melt state and the active ingredient. Finally, the compounded EVOH—methyl anthranilate formulations were transformed into films of 50 microns in thickness and 10 cm in width using an extrusion roller calender line. Samples were stored in aluminium foils prior to characterization to prevent an undesirable release of the volatile oil.

### 2.3. Characterization of Active Films

#### 2.3.1. Identification and Quantification of Methyl Anthranilate in EVOH-Based Films

The presence of methyl anthranilate in EVOH-based films after the extrusion process was qualitatively confirmed by Fourier transform infrared spectroscopy in attenuated total reflectance mode (ATR-FTIR), using a TENSOR 27 Spectrophotometer (Bruker, Massachusetts, MA, USA) equipped with a diamond ATR sampling accessory. A background spectrum was acquired before the analysis to compensate the CO_2_ and moisture effect. The spectra of EVOH-based film formulations were then recorded in the 4000–700 cm^−1^ wavenumber range, averaging 64 scans and 4 cm^−^^1^ resolution.

The final content of MA remaining in the active films was quantitatively determined by solid–liquid extraction followed by gas chromatography mass spectrometry (GC/MS) analysis. In brief, 1 cm^2^ of each film formulation containing methyl anthranilate was extracted with 5 mL of ethanol at 40 °C for 24 h. Then, GC/MS analysis was performed employing a triple quadrupole GC/MS instrument from Agilent Technologies (Agilent Technologies, Inc., Santa Clara, CA, USA) equipped with an Agilent DB-624 30 m × 0.32 mm, 0.18 µm column, using hydrogen at 3.5 mL/min as carrier gas with a 10:1 split ratio. For the analysis, 1 µL of the extraction in ethanol was injected. The GC oven was programmed at 85 °C for 1 min and then the temperature was increased by 30 °C min^−1^ to 220 °C, where it was held for 1 min. The quantitative determination of methyl anthranilate was conducted by using a full scan between the m/z values 40–200 and total ion counting. The calibration curve with a R^2^ > 0.99 was prepared by injecting solutions of methyl anthranilate in ethanol at five known concentrations into the GC/MS instrument. Then, the capacity of EVOH copolymer to retain the active methyl anthranilate compound was determined by employing the following Equation (1) [32].

(1) $$\mathrm{Retention}\;\mathrm{capacity}\;\left(\%\right)=\frac{m_{f}}{m_{i}}\times 100$$

where *m_f_* is the amount of methyl anthranilate in the packaging material after the extrusion process determined by GC/MS (g methyl anthranilate/g film) and *m_i_* is the nominal content of the active compound initially incorporated to the EVOH copolymer.

#### 2.3.2. Morphological Characterization 

The sample surfaces and cross sections, previously coated under vacuum conditions, using a current of 12 mA and for two minutes with a Pd/Au layer, were studied by employing a Hitachi S-4800 (Hitachi Ltd., Tokyo, Japan) microscope operated at 10 kV and a working distance of 10 mm.

#### 2.3.3. Thermal Analysis

The thermal stability and the degradation behaviour of EVOH-based film formulations was evaluated by thermogravimetric analysis (TGA) using a TGA Q-500 thermogravimetric instrument (TA Instruments, New Castle, DE, USA). Typically, 3–8 mg of film sample was placed in alumina pans and then heated from room temperature to 900 °C at a constant heating rate of 10 °C min^−1^ under N_2_ atmosphere with a flow rate of 50 mL min^−^^1^.

The determination of the main thermal parameters and transitions was performed by differential scanning calorimetry (DSC). DSC analyses were carried out by employing a DSC Q-2000 equipment from TA Instruments (New Castle, DE, USA). Approximately 5 mg of each sample was introduced in a sealed 40 µL aluminum pan and was subjected to the following thermal cycles: first heating scan from 0 °C to 200 °C at 10 °C min^−1^, followed by a cooling to −25 °C and a second heating scan to 200 °C. The temperature was held for 3 min at the end of each cycle. The values of the glass transition temperature, the melting and crystallization temperatures and their corresponding enthalpies were calculated with the TA Universal Analysis software by analyzing the DSC thermograms. The degree of crystallinity was then calculated as described elsewhere [33], taking 217.8 J g^−1^ as the melting enthalpy associated with a pure crystalline EVOH composed of 44% ethylene molar content and following Equation (2) [34].

(2) $$\chi \left(\%\right)=\frac{1}{\left(1-m_{f}\right)}\;\left[\frac{\Delta H}{\Delta H_{0}}\right]\times 100$$

where ΔH is the enthalpy for melting or crystallization; $\Delta H_{0}$ is melting for a 100% crystalline EVOH sample and $\left(1-m_{f}\right)$ is the weight fraction of EVOH in the sample.

#### 2.3.4. Mechanical Properties

The tensile characteristics of all films were calculated with an MTS Universal Tensile test machine (MTS Systems Corporation, Minnesota, MN, USA) equipped with a 1 kN load cell. The main mechanical properties (i.e., Young’s modulus (E), stress at yield (σ_y_), stress at break (σ_b_) and elongation at break (ε_b_)) were calculated from the stress-strain curves following UNE-EN ISO 527-3:2019 [35]. The dimensions of the film specimens were 170 mm long, 15 mm wide and 50 µm thick and the clamps distance was set at 100 mm. The films were stretched at 100 mm min^−^^1^ until failure. At least five measurements were conducted of each set of formulations and the results were expressed as the average ± standard deviation. A statistical analysis at a 5% significance level based on the ANOVA test was employed to analyse the results.

#### 2.3.5. Film Thickness, Colour Tests and Transparency

The film thickness of the films was determined employing a micrometre MiniTest 7200FH (ElektroPhysik Dr. Steingroever GmbH & Co, Köln, Germany) with a low range resolution of 0.1 µm. The mean values were calculated from readings taken at 10 different random locations of each film formulation. 

The change in the colour properties on EVOH films provoked by the incorporation of the active agent was evaluated by employing a KONICA CM-2500d (Konica Minolta Sensing Americas, Inc., Ramsey, NJ, USA). The apparatus was calibrated using a white and black standard tile. The tests were run in triplicate in the CIELab space, where L*, a* and b* indicate lightness, redness/greenness, and yellowness/blueness, respectively. The measurements were taken at random positions over the surface of the films and the results were expressed as the average values of at least three measurements. The total color difference (ΔE*) caused by methyl anthranilate in EVOH-based films was calculated following Equation (3).

(3) $$\Delta E={\left(\Delta L^{2}+\Delta a^{\;*2}+\Delta b^{\;*2}\right)}^{0.5}$$

where ΔL*, Δa*, and Δb* are the distances from the color coordinates of film samples to control EVOH film [36].

The light transmittance of the films was evaluated in the wavelength range of 400 to 800 nm using a Jasco V-630 UV-vis spectrophotometer (Jasco Deutschland GmbH, Pfungstadt, Germany). The transparency of the films was calculated following Equation (4)

(4) $$\mathrm{Transparency}\;=\frac{A_{600}}{t}$$

in which A_600_, is the absorbances at 600 nm, and t is the film thickness in millimetres [37].

#### 2.3.6. Barrier Properties

The water vapour permeability tests (WVP) were carried out at room temperature and at two different relative humidity (RH) levels, 33% and 75% RH, following the gravimetrically method based on the standard ASTM E96M [38]. Permeability cells were filled with 4 grams of the desiccant anhydrous calcium chloride, which was dehydrated at 90 °C for 48 h before the tests. Circular shaped film samples with a diameter of 5 cm were fixed to the cell with a flat silicon ring, leaving a permeable surface of 8 cm^2^. Then, the permeation cups were stored at room temperature in chambers containing saturated salt solutions, magnesium chloride, MgCl_2_, and sodium chloride, NaCl, for 33 and 75% RH, respectively. The weight gain of the cups over time was used to determine the water vapour transferred through the films and absorbed by the anhydrous calcium chloride. Cups were weighted for 21 days with an analytical balance and the water vapour transmission rate (WVTR) was calculated from the plot of the weight increment vs. time following Equation (5).

(5) WVTR (g h−1m−2)=ΔmΔtA

where Δ*m/*Δ*t* is the slope of the curve and *A* is the permeable surface of the film [39]. The obtained WVTR values were then divided by the water pressure gradient and multiplied by the film thickness to determine the water vapour permeability (WVP). Five replicates were tested for each set of formulations.

#### 2.3.7. Influence of Environmental Humidity on the Release of Methyl Anthranilate

In order to study the influence of humidity on the compound release, an extraction technique and GC/MS analyses were performed. Film samples of each formulation were conditioned at three different humidity values, 33%, 75% and >95% at room temperature for 10 days based on the methodology proposed by Kurek and co-workers [40]. After that time, films were recovered from their conditioning chambers, and the remaining amount of methyl anthranilate in the samples was extracted with ethanol. Then, GC/MS was carried out following the procedure described in Section 2.3.1 and the percentage release of methyl anthranilate was calculated as follows:

(6) $$\mathrm{Compound}\;\mathrm{release}\;\left(\%\right)=\frac{c_{i}-c_{f}}{c_{i}}\times 100$$

where *c_i_* is the real concentration of methyl anthranilate in the films (mg MA/g film) after the extrusion process, and *c_f_* is the final amount of methyl anthranilate in the films after its release promoted by humidity.

### 2.4. Efficacy of EVOH Films Containing Methyl Anthranilate against Fungal Growth In Vitro

#### 2.4.1. Microbiological Studies

*Penicillium expansum* (CECT 2278) and *Botrytis cinerea* (CECT 2100) were chosen as fungal models of foodborne pathogens. These strains were obtained from the Spanish type culture collection (CECT). Both fungi were grown and maintained in Potato Dextrose Agar (PDA, Scharlab, Spain) for 7 days at 26 °C. To carry out the antimicrobial assay, a conidial suspension of 10^6^ spores/mL was obtained from of the fungal surface of PDA plates. For this propose, sterile peptone water with 0.05% (*v/v*) Tween 80 was poured on the fungal plate and scratched with a Digralsky spreader to drag the conidia. The fungal culture solution was transferred to sterile tubes and several dilutions were carried out to obtain the concentration of 10^6^ spores/mL, counted with improved Neubauer chamber (Bright-Line Hemacytometer, Hausser Scientific, Horsham, PA, USA).

#### 2.4.2. Antifungal Activity of Methyl Anthranilate

The study of the antifungal capacity of the methyl anthranilate (MA) in vapour phase against *Penicillium expansum* and *Botrytis cinerea* was carried out by determination of values of minimal inhibitory concentration (MIC) and minimal fungicidal concentration (MFC). The MIC value was defined as amount of compound which inhibits growth by at least 50% compared to the control at day 7 of incubation. MFC was defined as the amount of volatile that completely inhibit the fungal growth after 10 days. Both parameters have been expressed as volume (μL) of active compound dosed in each inoculated plate. The PDA plates were inoculated in three equidistant points with 3 μL of conidia suspension previously obtained (10^6^ spores/mL). Then, a volume of 1, 2.5, 5, 10, 20, 50 μL of MA was placed in a 50 mm of sterile paper disk and fixed in the lid of the plate. The plates were closed and sealed with parafilm to avoid the volatile loss and incubated during 7 days at 26 °C. The fungal colony diameter was measured after that time to determinate the MIC value (μL/plate). Then, the active compound was removed from the plates and were incubated for 3 days more, and fungal growth was assessed to determinate MFC parameter (μL/plate). All assays were carried out in triplicate.

#### 2.4.3. Antifungal Activity of Cast-Extruded EVOH Films Incorporated with Methyl Anthranilate

Assessment of the antimicrobial activity of cast extruded EVOH films incorporated with methyl anthranilate against both fungal strains was carried out by micro-atmosphere assay, which is based on the effectiveness of the volatile vapour phase generated inside the plate. To conduct this assay, 0.3 g of each film of 8 cm of diameter, with different amount of MA (0, 3, 5, 8 wt.%), was placed on the lid of the petri dish, which contained 15 mL of PDA and was inoculated at 3 equidistant points with conidial suspension. The plates with active film and fungal inoculation were incubated for 10 days at 26 °C. The growth of the fungal colony was monitored by measuring colony diameter at 3, 5, and 7 days, after which the film was removed and incubated for 3 days more to observe the recovery of the fungal colony. A control without film was also carried out. The radial growth of colony was measured in cm and inhibition of fungal growth in respect to the control was calculated and expressed as percentage. All assays were conducted in triplicate.

## 3. Results and Discussion

### 3.1. Identification and Quantification of Methyl Anthranilate in EVOH-Based Films

**Figure 2:**
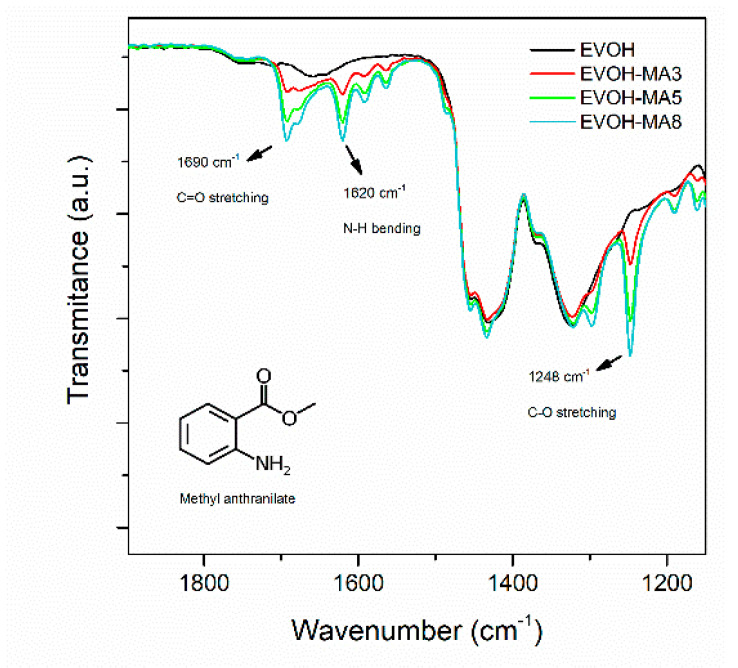
FT-IR spectra of EVOH-MA samples.

The FTIR spectra in the region of interest of EVOH and EVOH active films are shown in Figure 2. The spectrum of the control EVOH film showed bending C-H vibrations at 1328 cm^−^^1^ and 1452 cm^−^^1^, and the absorbance bands related to stretching vibrations located at 2850 cm^−^^1^ (C-H), 2930 cm^−^^1^ (C-H) and 3342 cm^−^^1^ (O-H) (not shown in the Figure 2). The presence of a significant amount of methyl anthranilate in EVOH copolymer matrix after the extrusion process was confirmed by the observation of several absorbance peaks related to the bioactive compound such as the C=O stretching vibration band at 1690 cm^−^^1^, the bending of the N-H at 1620 cm^−^^1^, and by the C-O stretching vibration at 1248 cm^−^^1^ [41]. Moreover, the intensity of these peaks augmented as the concentration of methyl anthranilate increased in the active extruded films.

To determine the real concentration of MA in the polymeric EVOH films, GC/MS analysis was performed via a previous extraction with ethanol at 40 °C. The final concentrations for each formulation are displayed in Table 1. As shown, the final content of MA in the films was below their initial content, possibly due to losses occurred in the extrusion process caused by the high temperatures and the shear stresses generated during the film manufacturing. Results revealed that less than 25% of the content of MA was lost during processing, indicating a high retention capacity of the volatile oil in the polymer matrix. The used screw design, which favoured a proper mixing of the components, together with the extrusion conditions were the main factors that permitted a considerable amount of the antifungal compound to remain in the material after the processing. Other authors working with volatile organic compounds intended for active food packaging, such as carvacrol, reported a retention capacity lower than 50% when this substance was incorporated into polypropylene by melt extrusion [42]. Considering thymol (THY), another widely employed volatile compound with a boiling point similar to methyl anthranilate (232 °C THY vs. 256 °C MA) that has demonstrated antibacterial and antioxidant activities, a loss of nearly 30% was found by Ramos et al. when it was directly incorporated into poly(lactic acid) by melt processing [43]. Interestingly, they reported that the amount of thymol after the extrusion process was slightly higher in the formulations containing a nanoclay, indicating that the presence of this additive can retard the compound evaporation during the manufacturing process. A similar behaviour was also observed by the incorporation of Ag nanoparticles in PLA/THY formulations produced by melt compounding [44]. A significantly higher loss of thymol was observed by Galotto and co-workers, who reported a loss of 70% when this volatile compound was incorporated with polyethylene, owing to the high temperatures and to the low chemical compatibility between the two components [45]. Therefore, based on the obtained results, it is noticeable that the employed screw configuration and extrusion conditions may have played a crucial role to consider methyl anthranilate a good antifungal compound for the preparation of active packaging films by the melt blending processes.

### 3.2. SEM Analysis

**Figure 3:**
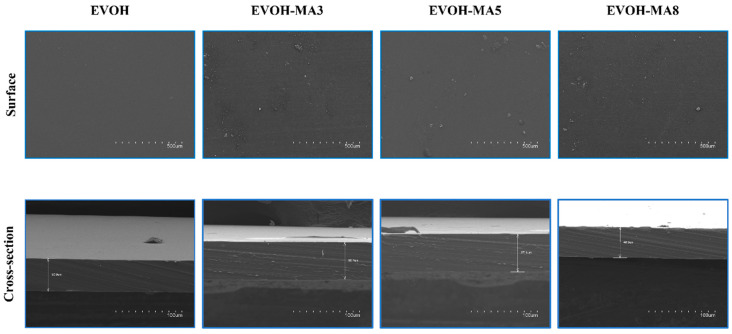
SEM micrographs of sample surfaces and cross sections (×100 and ×500, respectively.).

Figure 3 displays the SEM pictures acquired for the surface and cross section of the film samples. The surface of EVOH control film showed, as expected, a clean and homogeneous surface. Regarding the films containing methyl anthranilate, smooth surfaces could be observed for the three active formulations. However, some pores and imperfections could be detected in certain regions along the examined area. This observation may be attributed by an eventual loss of the active ingredient present in the surface of the material after the manufacturing process [46]. On the other hand, the cross sections of the films subjected to cryofracture, revealed a smooth and non-porous morphology, with no appreciable phase separation in the films containing methyl anthranilate, which indicated a good dispersion and compatibility between the polymer matrix and the natural ingredient. In addition, a film thickness around 50 µm could be confirmed for all formulations by SEM observations.

### 3.3. Thermal Properties

**Figure 4:**
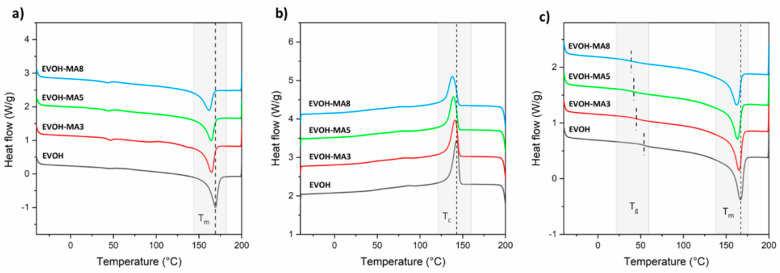
DSC thermograms during (**a**) first heating, (**b**) cooling and (**c**) second heating scan.

The DSC thermograms of the different cast-extruded EVOH-based films, corresponding to the heating and cooling stages, are displayed in Figure 4 while the main thermal parameters obtained from the analysis of the DSC curves, are reported in Table 2. In the control EVOH film, it was possible to observe during the first heating scan a second-order transition related to the glass transition temperature of EVOH copolymer, with a value of 52 °C, determined as the midpoint between the onset and the end of the inflectional tangent.

Then, an endothermic process related to the melting of EVOH copolymer was observed at a peak temperature around 162 °C, with a ∆H_m_ of 68.8 J/g, resulting in a degree of crystallinity of 31.5%. With respect to the bioactive EVOH films in which methyl anthranilate was incorporated, the glass transition temperature values decreased in temperature, ranging between 37 and 43 °C, indicating an additive-induced plasticization over the EVOH copolymer. This phenomenon was more evident with the highest incorporated amount of MA in the films, that is, 8 wt.%, resulting in a decrease in the T_g_ of 15 °C. The addition of the active ingredient also shifted the melting peak towards lower temperatures with respect to the control EVOH sample. Specifically, the T_m_ of pristine EVOH was reduced by 4 °C, 5 °C and 7 °C for the 3, 5 and 8 wt.% MA content, respectively. Moreover, the melting enthalpy was seen also to decrease when increasing the MA content, possibly due to alterations in the crystalline structure of EVOH copolymer, and, as a consequence, the degree of crystallinity was reduced from 31.5% to 26.4%, 27.3%, and 25.4% for the film formulations containing 3, 5 and 8 wt.% of MA, respectively. This effect has been observed in previous studies dealing with EVOH copolymer, and was related to interactions occurring between hydroxyl groups present along the copolymer structure and low molecular weight compounds in the amorphous phase [47,48]. Considering the cooling step from the melt (Figure 4b), it was possible to observe, as expected and in good agreement with the heating scan observations, that pristine EVOH presented a crystallization process with a peak temperature centred at 143 °C and a crystallization enthalpy (∆H_c_) of 61.8 J/g. As reported in the literature, depending on both, the content of vinyl alcohol in the copolymer and the cooling rate from the melt state, EVOH copolymers family is able to crystallize in two types of phases: orthorhombic or monoclinic [49,50]. In the case of EVOH_44_, Cerrada et al. concluded that this particular copolymer crystallized, independently of the cooling rate, in the orthorhombic phase [51]. With regard the active films, both, the T_c_ and ∆H_c_, decreased proportionally with the content of MA in the samples. Therefore, it is possible to assume that the presence of the active compound slightly hinders the correct ordering of the copolymer chains, needing higher undercoolings to crystallize and, consequently, shifting the T_c_ to lower values. During the second heating cycle, the thermal parameters obtained for pristine EVOH were similar to those obtained during the first heating scan. However, the T_g_ values reported for the active samples were slightly higher when compared to the first heating scan, possibly due to a potential loss of the active ingredient during the different heating and cooling cycles.

The results obtained in the present study are consistent and well supported by those reported in previous works found in literature for compounded EVOH-based materials developed by melt-extrusion and other manufacturing techniques (i.e., solvent casting, electrospinning, etc.) For instance, Luzi et al. reported a decrease in the T_g_, T_m_ and χ_c_ of EVOH-caffeic acid-based systems prepared by solvent-casting [48]. In another work, Luzi and her co-workers studied the effect of the processing technique, solvent casting and extrusion processing on the properties of EVOH based films incorporated with gallic acid and umbelliferone. They observed a general decrease of the melting temperature, melting enthalpy and degree of crystallinity caused by the presence of the active ingredients [52]. More recently, Meléndez et al. developed electrospun EVOH_44_ fibres with and without cellulose nanocrystals (CNC), and they found lower T_c_, T_m_ and χ_c_ values in the samples containing CNC compared to the pristine copolymer [9].

**Figure 5:**
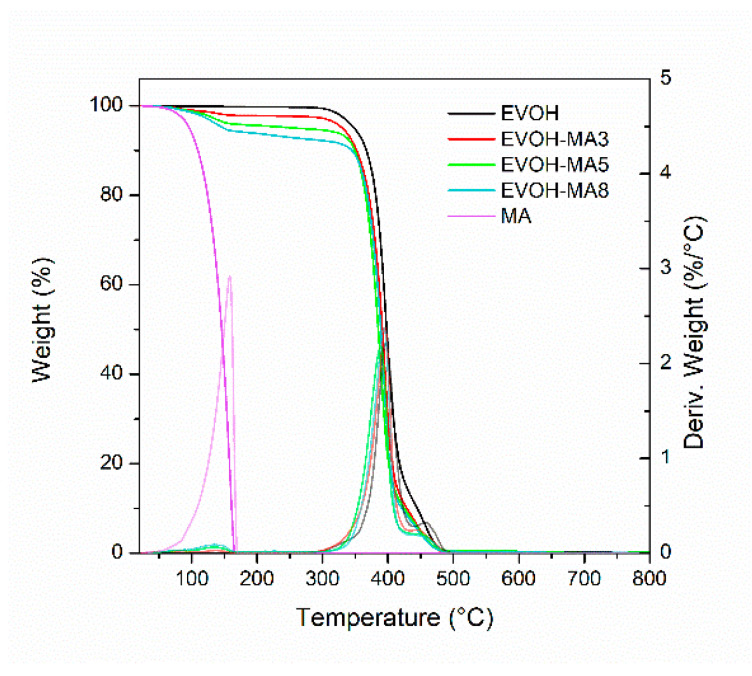
TGA and DTG curves for EVOH-based samples.

With respect to the thermal stability of the samples, EVOH control film presented two major weight loss zones at 300–420 °C and 420–485 °C, corresponding to 90% and 9% mass loss, respectively. The first decomposition step in EVOH film was related to the degradation reactions occurring in the vinyl alcohol component in the copolymer while the second degradation stage, with a maximum peak temperature centred at 459 °C, was associated with the full decomposition of EVOH backbones [53,54]. Films incorporating 3–8 wt.% methyl anthranilate showed a thermal degradation process located in the range of 120–200 °C (see Figure 5), indicating that the thermal volatilization of MA occurs in this temperature interval. As shown in Table 2, the weight loss increment (ΔW_0–200_) in this range was in good agreement with both the nominal amount of methyl anthranilate incorporated in the films during the cast-extrusion process and the results extracted from the GC-MS analysis. Interestingly, the maximum peak degradation temperatures (T_maxI_ and T_maxII_) of EVOH copolymer were not significantly affected by the presence of methyl anthranilate, indicating that film formulations maintained a high thermally stable behaviour. Similar results have been observed in previous studies where natural volatile compounds were blended with other thermoplastic polymers [46,55].

### 3.4. Mechanical Properties

**Figure 6:**
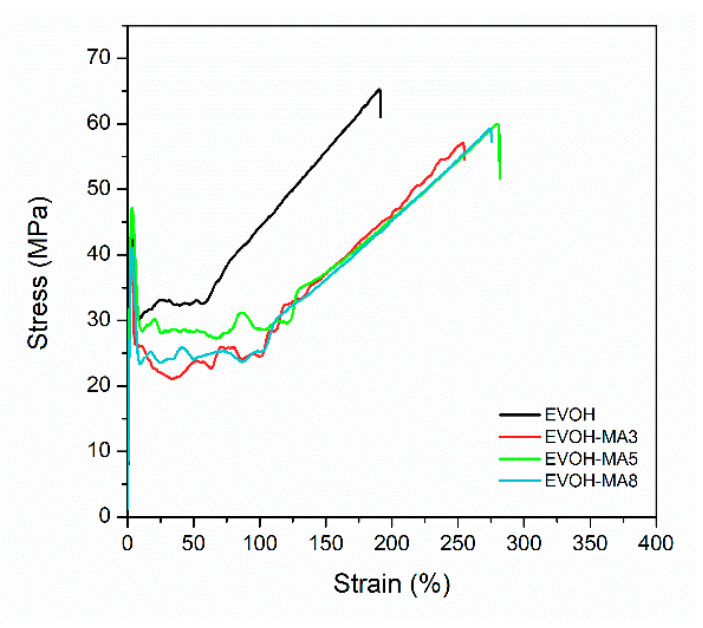
Representative stress-strain curves of EVOH-based films.

Figure 6 presents the tensile stress-strain curves of the cast-extruded films from which the mechanical properties, i.e., Young Modulus (E), stress at yield (σ_y_), stress at break (σ_b_) and elongation at break (ε_b_) values were calculated. Incorporation of methyl anthranilate slightly decreased the Young Modulus from 2.5 ± 0.2 GPa of EVOH control film to a value of 2.2 ± 0.1 GPa of EVOH film incorporating up to an 8 wt.% of methyl anthranilate, indicating that the bioactive film formulations had less rigid behaviour. After reaching the yield point, it was possible to observe a drop of the stress values, pointing out a necking effect in EVOH-based formulations. The plasticization produced by methyl anthranilate could explain the decreasing tendency in the yield stress from 46 ± 3 MPa in EVOH film to 40 ± 5 MPa in EVOH-MA8 film samples [56]. Beyond the yield point, a strengthening behaviour during the plastic deformation—strain hardening—was detected for all films.

This phenomenon, caused by the orientation and alignment of polymer chains, was more remarkable in neat EVOH, owing to its higher degree of crystallinity with respect to bioactive films, as determined in DSC analysis [57]. Consequently, the stress at break value of EVOH film (62 ± 5 MPa) was higher when compared of films incorporating methyl anthranilate, whose values decreased as the content of the active substance increased in the EVOH-based films. Finally, the plasticizing effect of methyl anthranilate over the EVOH matrix resulted in a noticeable increase in the elongation at break values, shifting from 174% in EVOH film to 253% in EVOH-MA8 formulation. A similar behaviour has been reported in previous works in which essential oils or their active constituents were incorporated in thermoplastic polymers by melt processing techniques [58,59,60]. In this line, the mechanical performances of all film formulations showed their suitability for flexible packaging applications.

### 3.5. Water Vapour Permeability

The WVP was tested at 33% and 75% relative humidity at room temperature and the values are gathered in Table 3. Regarding the influence of RH on the WVP, all samples showed the same tendency: the permeability was significantly higher for the samples measured at 75% RH, as a consequence of the plasticizing effect of water over the EVOH copolymer chains. The increase in the polymer chain mobility results in a reduction in the cohesive energy in the -OH groups present in EVOH, thus accelerating the molecular diffusion of sorbed water through the film [61]. In particular, the WVP of pure EVOH_44_ shifted from a value of 0.12 × 10^−^^15^ kg·m m^−^^2^ s^−^^1^ Pa^−1^ at 33% RH to 1.08 × 10^−15^ kg·m m^−^^2^ s^−^^1^ Pa^−1^ at 75%, which are in good concordance with those reported in previous works [9,62]. The incorporation of 3 wt.% of methyl anthranilate slightly reduced the WVP with respect to the control EVOH film at both tested humidity values, possibly due to the low affinity and sensitivity of the active ingredient to water. However, films containing 5 and 8 wt.% of MA seemed to be more water sensitive than the control EVOH film and EVOH-MA3 samples at 33 and 75% RH. This result can be explained as a consequence of the significant plasticizing effect observed in the thermal analysis for the active films and the resulting increase in the amorphous region suitable for water transport in these samples.

### 3.6. Optical Properties 

The visual aspect of packaging films is a key factor since it can influence the consumer perception on a certain end-product. In this line, Table 4 reports the main optical and colour parameters of the cast-extruded active films based on EVOH copolymer incorporated with methyl anthranilate, which all had an average thickness in the range of 50–55 µm. EVOH control film was characterized by a high transparency and with the addition of MA, the film formulations maintained their original appearance. The developed films exhibited very similar *L* values, 92–93, indicating a high lightness of the colour of the active films. This was attributed to a good dispersion of the active compound in the polymer matrix. Furthermore, the colour parameter a* of EVOH copolymer film, which indicates the green (negative) or red colour (positive), was not significantly affected by the presence of methyl anthranilate in the active formulations, shifting towards negative values as the content of the active ingredient augmented in the films. On the contrary, a clear tendency to positive values of b* parameter provoked by the incorporation of methyl anthranilate was observed in the active EVOH-MA films. Therefore, these samples showed a slight yellow tonality that was responsible for the colour change (ΔE) in EVOH-MA formulations, as reported in the Table below. The total colour difference was in the range of 4.1–4.9, which means that the deviation in the optical properties can be noticeable by an unexperienced observer [34]. On the other hand, in terms of transparency, it was observed that the T parameter slightly decreased by increasing the MA content. Thus, EVOH film and EVOH sample with 3 wt.% of MA showed *T* values of 0.89 and 0.88, respectively, but by increasing the MA content up to 8 wt.%, the transparency value was seen to decrease more evidently (0.77). With all this, it is possible to state that the presence of methyl anthranilate did not significantly affect the great optical properties of EVOH copolymer film, since the colour deviations were moderate and the transparency in the active films remained practically unchanged with respect to the control EVOH film, demonstrating the potential applicability of the developed active films for food packaging.

### 3.7. Effect of Environmental Humidity on Methyl Anthranilate Release from EVOH-Based Films

**Figure 7:**
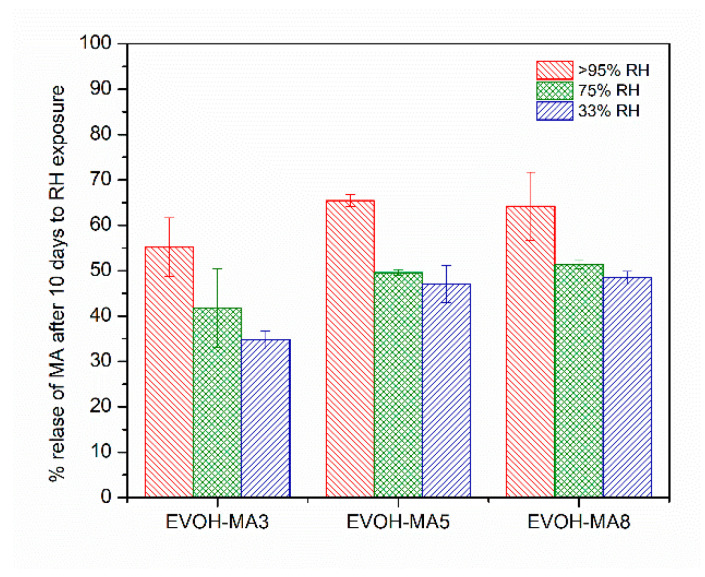
Methyl anthranilate release from EVOH based films after 10 days influenced by relative humidity (33, 75 and >95%).

One of the main factors that influences the success for developing active packaging materials is related to the trigger for the release of the active from the polymer matrix to achieve a sustained release to the food media. As commented before, EVOH copolymers are characterized by a hydrophilic nature due to their hydroxyl content. Therefore, as the EVOH-based films are exposed to different relative humidity, water molecules begin to penetrate through the polymer structure, leading to a swelling of the matrix, thus enabling the release of methyl anthranilate. As displayed in Figure 7, the % release was greatly influenced by the hydration of the system, with the lowest at 33% RH and the highest at >95% RH. At 33% RH, less than 50% methyl anthranilate was released from the three active formulations. On the other hand, in the highest humidity environment, the release of the active ingredient from the polymer matrix to the head space of the system reached a 65% after 10 days of exposure, associated with the sorption of water in the material and the subsequent hydration of the polymer structure. This result indicated that a significant amount of MA was still present and available to be further released from the active films after ten days of exposure to different RH. This observation was attributed to the fact that EVOH-based films may need longer times to reach equilibrium, as reported by Aucejo and co-workers [63]. In this line, a longer interaction between water and EVOH based films may lead to a greater plasticization and swelling of the polymer matrix and thus to a greater release of methyl anthranilate. In a work conducted by Kurek et al., the effect of relative humidity on carvacrol release from chitosan-based films was intensely studied [40]. They reported that at 0% relative humidity and after 60 days at room temperature, the remaining amount of carvacrol was higher than 45%, while at >96% RH, already after 10 days more than 95% of the carvacrol was released. The results presented herein show that the developed active films may have a great potential to be applied in food products with high water activity and thus where a high relative humidity is generated, permitting the release of methyl anthranilate, and, consequently, inducing the potential antimicrobial effect over the food produce.

### 3.8. Antifungal Activity

#### 3.8.1. Methyl Anthranilate Vapour Inhibition Assay

The antimicrobial activity of methyl anthranilate (MA) against *Penicillium expansum* and *Botrytis cinerea* was evaluated through micro-atmosphere generated into fungal culture plates. The effectiveness of volatile compound expressed as MIC and MFC (μL/plate) are represented in Table 5. Both fungi showed a great inhibition when MA was applied in the plate. *B. cinerea* showed the highest susceptibility to a low amount of MA; thus, 5 μL of active compound exhibited a fungicidal activity. However, a higher amount of compound is required to reach total inhibition of *P. expansum*. Few studies have reported in the literature on the antimicrobial activity of MA. Nidiry and Babu, (2005) evaluated the antifungal effect of some tuberose constituents, including methyl anthranilate, which showed good effectiveness against *Colletotrichum gloeosporioides* [64]. Nevertheless, the antifungal activity was assessed through diluting the compound in the agar culture medium, and not as the vapour phase. Other studies have also reported the antifungal activity of the active ingredient against fungi food pathogens applied as vapour phase, demonstrating the good effectiveness of MA [65,66]. The previous studies and the obtained values for MIC and MFC in the present research (μL/plate) against *P. expansum* and *B. cinerea*, highlight the potential of this compound for its incorporation in active food packaging systems.

#### 3.8.2. Antifungal Activity of Cast-Extruded Active EVOH-Based Films

**Figure 8:**
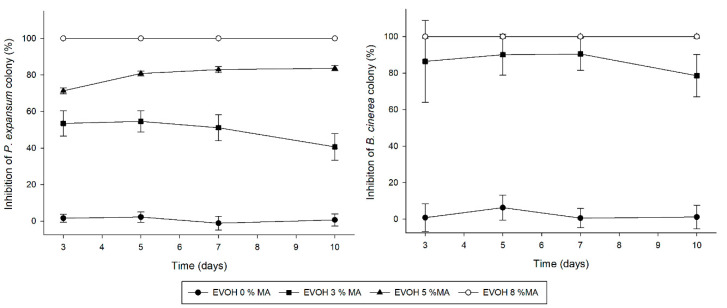
Effectiveness of EVOH incorporated with methyl anthranilate against *P. expansum* and *B. cinerea* expressed as inhibition of fungal colony (%) during 10 days of at 26 °C.

The effectiveness of active films containing methyl anthranilate with 3, 5 and 8 wt.% was evaluated against both fungi by the generation of a micro-atmosphere inside petri dishes. The inhibition of fungal colony was calculated with films containing different amount of MA, and is depicted in Figure 8. As discussed above, EVOH copolymer is a highly hydrophilic material due to its great hydroxyl group content, increasing aroma permeation in presence of high relative humidity. The EVOH-based films in the petri dish were subjected to a high-humidity environment, which allowed MA to be released from the polymer matrix to the plate headspace, allowing it to exert its activity. In good agreement with the obtained data of MIC and MFC, it could be observed that the active films exhibited great effectiveness against *B. cinerea*. Thus, fungicidal activity was observed for films with 5 and 8 wt.% of MA. Moreover, films with 3 wt.% of MA showed an inhibition around 80% during all days of incubation.

**Figure 9:**
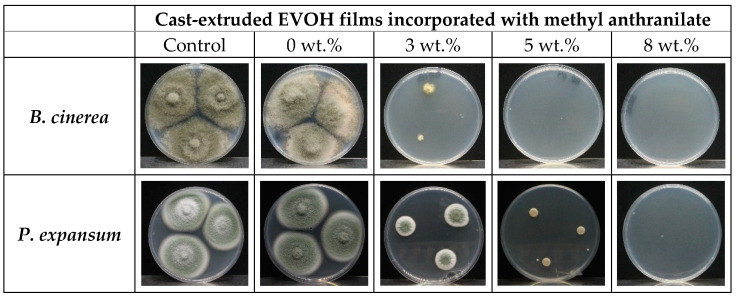
Visual aspect of fungal colony of *P. expansum* and *B. cinerea* co-incubated with cast extruded EVOH films incorporated with methyl anthranilate after 7 days at 26 °C.

On the other hand, EVOH film incorporated with the highest amount of MA (8 wt.%) also resulted in fungicidal activity against *P. expansum*, while this fungi incubated in the presence of EVOH-MA3 and EVOH-MA5 exhibited a great inhibition, and colonies resulted that were white and small, which could be associated with an inhibition of sporulation [67]. The visual aspect of fungal colonies after seven days of incubation is represented in Figure 9, which shows the great effectiveness of the developed active films against both fungi. The antimicrobial activity of thermo-processed films may be compromised by losses occurring during film processing [55,68]. In our study, cast extruded EVOH films incorporated with MA showed great antifungal activity due not only to the effectiveness of MA, but also to the high retention capacity of the active ingredient in the films after the manufacturing process. Thanks to the hydrophilic nature of EVOH copolymer, the compound release from the polymer matrix is mainly due to the exposition of films to high relative humidity since fungal growth medium produces a high humidity atmosphere in plates, which leads to a rapid release of the volatile substance, allowing strong inhibition of microorganisms [5].

Several studies have previously incorporated essential oils in EVOH copolymer matrix aiming to develop antimicrobial polymers with interest in food packaging [5,13,69,70,71]. Although, to the best of our knowledge, no studies have been reported in which methyl anthranilate is incorporated into a polymer matrix intended for packaging applications, its antifungal activity on fruit pathogens has been previously demonstrated [65,66]. According to these studies and the great fungal inhibition rates of EVOH-based films with MA presented in this work, even with the minor content of the ingredient, the developed films show a great potential to be applied in active food packaging.

## 4. Conclusions

In the present work, novel active EVOH-based films containing 3 wt.%, 5 wt.%, and 8 wt.% of bioactive methyl anthranilate compound were prepared at pilot scale by melt extrusion processing in a twin-screw extruder with a specifically designed screw configuration, and fully characterized. All active formulations presented a retention capacity of the natural ingredient higher than 75%. Films were optically transparent, but methyl anthranilate provoked a somewhat yellow tonality. DSC analysis indicated a plasticizing effect of MA onto the EVOH matrix, resulting in a decrease in the glass transition temperature of up to 15 °C, while the thermal stability of EVOH-based films evaluated by TGA was not significantly affected by the presence of the antifungal ingredient. The incorporation of different concentrations of methyl anthranilate into EVOH-based films resulted in an increase of the elongation at break values, from 174% in neat EVOH to 253% in EVOH-MA8 formulation, confirming the plasticizing effect of this compound. This behavior explained the slight increase in the water vapor permeability values in the film formulations containing high amounts of methyl anthranilate (5 and 8 wt.%) with respect to the pristine EVOH film. Despite this, all active films showed positive results concerning antifungal activity, exhibiting fungicidal activity against *B. cinerea* and *P. expansum*. The results presented and discussed in this study show the feasibility of the used technique to develop active films with antifungal properties scalable in industrial environments. 

## Figures and Tables

**Table 1:** Film formulations and retention capacity of methyl anthranilate quantified by GC-MS analysis.

Formulation	Code	Methyl Anthranilate (wt.%)	Retention Capacity (%)
Neat EVOH	EVOH	n.d.	-
EVOH + 3 wt.% methyl anthranilate	EVOH-MA3	2.52 ± 0.36	84
EVOH + 5 wt.% methyl anthranilate	EVOH-MA5	3.84 ± 0.20	76
EVOH + 8 wt.% methyl anthranilate	EVOH-MA8	6.42 ± 0.17	80

**Table 2:** DSC and TGA parameters.

	First Heating Scan				Cooling Scan		Second Heating Scan				TGA		
Formulation	T_g_ (°C)	T_m_ (°C)	∆H_m_ (J/g)	χ_c_ (%)	T_c_ (°C)	∆H_c_ (J/g)	T_g_ (°C)	T_m_ (°C)	∆H_m_ (J/g)	χ_c_ (%)	ΔW_0–200_ (%)	T_maxI_ (°C)	T_maxII_ (°C)
EVOH	52	169	68.8	31.5	143	61.8	54	167	66.5	30.5	0.2	399	459
EVOH-MA3	43	165	55.7	26.4	141	56.8	44	165	65.3	30.9	2.25	395	445
EVOH-MA5	40	164	57.4	27.3	139	53.9	42	163	56.9	27.4	4.33	389	452
EVOH-MA8	37	162	50.9	25.4	138	52.9	39	162	52.7	26.3	6.12	392	451

**Table 3:** Mechanical properties and water vapor permeability values of EVOH-based films.

Reference	E (GPa)	Stress @ Yield (MPa)	Stress @ Break (MPa)	Elongation @ Break (%)	WVP·10^15^ (kg·m/m^2^ s Pa)	
					33% RH	75% RH
EVOH	2.5 ± 0.2 ^a^	46 ± 3 ^a^	62 ± 5 ^a^	174 ± 16 ^a^	0.12 ± 0.01	1.08 ± 0.02
EVOH-MA3	2.4 ± 0.1 ^a^	41 ± 4 ^a^	54 ± 7 ^b^	224 ± 18 ^b^	0.11 ± 0.01	0.99 ± 0.04
EVOH-MA5	2.3 ± 0.2 ^a^	41 ± 4 ^a^	46 ± 10 ^b,c^	260 ± 21 ^c^	0.15 ± 0.02	1.14 ± 0.02
EVOH-MA8	2.2 ± 0.1 ^a^	40 ± 5 ^a^	43 ± 8 ^c^	253 ± 12 ^c^	0.18 ± 0.01	1.38 ± 0.06

^a–c^: different superscript within the same column indicates significant different between film formulations (Turkey test, *p* < 0.05).

**Table 4:** Thickness, colour parameters and transparency of extruded EVOH-based films.

Reference	Thickness (µm)	L	a	b	ΔE	T (A_600_/t)
EVOH	50 ± 2	93.1	0.04	−0.49	-	0.89 ± 0.01
EVOH-MA3	53 ± 3	92.6 ± 0.1	−0.59 ± 0.02	3.6 ± 0.04	4.1 ± 0.1	0.88 ± 0.01
EVOH-MA5	55 ± 3	92.5 ± 0.1	−0.81 ± 0.01	4.12 ± 0.02	4.7 ± 0.2	0.84 ± 0.01
EVOH-MA8	54 ± 4	92.6 ± 0.1	−0.78 ± 0.05	4.31 ± 0.04	4.9 ± 0.1	0.77 ± 0.02

**Table 5:** Antimicrobial effectiveness of methyl anthranilate against *P. expansum* and *B. cinerea* expressed as MIC and MFC (μL/plate) after 10 days of incubation at 26 °C.

	MIC (μL/plate)	MFC (μL/plate)	Control	MIC	MFC
*P. expansum*	2.5	20	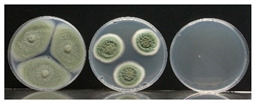		
*B. cinerea*	1	5	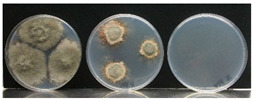		

## Data Availability

The data presented in this study are available on request from the corresponding author.
